# Microstructure, Properties, and Reversed Austenite Transformation Behavior of 04Cr13Ni5Mo Maraging Stainless Steel at Different Tempering Temperatures

**DOI:** 10.3390/ma19122440

**Published:** 2026-06-07

**Authors:** Hongru Lyu, Shoutai Rui, Yamin Peng, Xue Ji, Anhao Li, Deli Zhao, Qingxian Ma

**Affiliations:** 1 State Key Laboratory of Clean and Efficient Turbomachinery Power Equipment, Department of Mechanical Engineering, Tsinghua University, Beijing 100084, China; lv-hr23@mails.tsinghua.edu.cn (H.L.);; 2 China First Heavy Industries, Qiqihar 161042, China

**Keywords:** maraging stainless steel, reverse phase transformation, reverse austenite, mechanical properties

## Abstract

The influence of tempering temperature within the range of 520 °C to 640 °C on the microstructure and mechanical properties of 04Cr13Ni5Mo maraging stainless steel was systematically studied. The evolution of crystallographic orientation information, such as phase ratio and grain boundary ratio of the studied steel at different tempering temperatures, was studied by utilizing the electron backscatter diffraction (EBSD) technique. Furthermore, the element distribution at typical tempering temperatures was quantitatively analyzed by utilizing the electron probe microanalysis (EPMA) technique. Results indicated that the microstructure of the studied steel at different tempering temperatures is mainly composed of tempered sorbite. As the tempering temperature increased from 520 °C to 640 °C, the proportion of low-angle grain boundaries gradually increased while the proportion of large-angle grain boundaries decreased. The content of reversed austenite showed a sharp increase with the elevation of tempering temperature and peaked at approximately 9.0% at a tempering temperature of 640 °C. With the tempering temperature increasing from 520 °C to 640 °C, the strength of the studied steel showed a trend of first decreasing, then stabilizing, and then decreasing again, while the plasticity showed a stable upward trend. When the tempering temperature was 610 °C, the strength, plasticity, and toughness of the studied steel achieved the optimal match. The enrichment of the Ni element during the austenite reverse phase transformation process was confirmed as the predominant factor ensuring the stability of the reverse austenite to room temperature.

## 1. Introduction

As a typical grade of maraging stainless steel, 04Cr13Ni5Mo was developed on the basis of conventional martensitic stainless steel by reducing the carbon content, increasing the Ni content, and appropriately adding the Mo element. Owing to its excellent properties, including good corrosion resistance, wear resistance, high strength, and outstanding low-temperature impact toughness, this steel grade has been widely applied in fields such as the manufacturing of runners and guide vanes for hydraulic turbine units, as well as the transmission shafts of reactor auxiliary pumps [1,2,3]. Considering the alloy composition characteristics of 04Cr13Ni5Mo maraging stainless steel, the conventional heat treatment processes for this alloy steel are quenching and tempering. In order to obtain the optimal process control window for the mechanical properties of 04Cr13Ni5Mo, many researchers have carried out a series of research work focusing on the optimization of heat treatment processes. Zhi J.H. et al. studied the influence law of different tempering heat treatment processes on the microstructure and mechanical properties of 04Cr13Ni5Mo maraging stainless steel, and found that under the treatment process of 1050 °C × 1 h oil quenching followed by tempering within the range of 620–700 °C, with the increase in tempering temperature, the strength of the studied steel gradually increased while the plasticity and toughness decreased. Under the tempering condition of 700 °C, the specified non-proportional yield strength of the studied steel reached 937.5 MPa [4]. Zhang S.H. et al. [5] studied the influence of tempering time on reversed austenite and found that with the increase in tempering time, the filiform reversed austenite would transform into a granular shape, thereby reducing the stability of reversed austenite. Xie J.H. et al. studied the influence of quenching process on the microstructure transformation and mechanical property of 04Cr13Ni5Mo, and found that when quenching within the range of 940~1100 °C, with the increase in quenching temperature, the tensile strength and yield strength of 04Cr13Ni5Mo steel first decreased and then increased, and the yield strength reached the maximum value of 874 MPa under the quenching condition of 1100 °C [6]. Wu G.J. et al. [7] explored the influence of the quenching and tempering process parameters of 00Cr13Ni5Mo steel on its microstructure and found that the microstructure of quenched 00Cr13Ni5Mo steel was mainly composed of lath martensite and δ ferrite. When the quenching temperature exceeded 1100 °C, most of the δ ferrite presented a network distribution, and the strength of the material showed a downward trend. Sun T.H. et al. studied the influence of heat treatment process on the microstructure of the central part of large-size 04Cr13Ni5Mo forgings, and found that after the heat treatment of secondary normalizing (990 °C + 960 °C) + secondary tempering (550 °C + 590 °C), the grain size, impact absorption energy, and tensile properties of the tested forgings basically met the technical requirements [8]. Wang P. et al. [9] found that the existence of δ ferrite would significantly increase the ductile-brittle transition temperature of the material, which had an adverse effect on the toughness of the material. Zhang H.S. et al. found that after one normalizing and two tempering heat treatments, tempered sorbite structure was obtained, which could effectively avoid the generation of high-temperature δ ferrite, and the product performance could be improved [10].

Based on the above analysis, it can be seen that for materials of the same composition system of 04Cr13Ni5Mo, diversified control of mechanical properties can be realized by changing the heat treatment process. However, from the perspective of the strengthening and toughening mechanism of maraging hardened stainless steel, no matter what kind of heat treatment process is adopted for this type of steel, the essence of its strengthening and toughening is to obtain a structure with tempered sorbite or tempered troostite as the matrix through quenching and tempering processes. Combined with a certain amount of reversed austenite, the strengthening and toughening of the alloy are realized through the coupling effect of mechanisms such as dislocation strengthening, second-phase strengthening, and TRIP effect [11]. Therefore, systematically mastering the evolution law of the microstructure, especially the evolution law of reversed austenite, is of great significance for realizing the precise regulation of the mechanical properties of this type of maraging stainless steel. At present, there have been some reports on the microstructure evolution and the evolution behavior of reversed austenite of this type of maraging stainless steel [12,13,14]. Adopting the process of normalizing +steel and tempering, Li Z.G. et al. studied the microstructure and mechanical properties of 00Cr13Ni5Mo steel and found that with the increase in tempering temperature, the content of reversed austenite in the studied steel first increased and then gradually decreased, and the content of reversed austenite reached the peak when tempering at 600 °C [12]. Yang X.H. et al. investigated the influence of different tempering temperatures on the reversed austenite content of 04Cr13Ni8Mo2Al steel, and found that with the increase in tempering temperature, the reversed austenite content in the studied steel showed a change trend from basically unchanged to rapid increase and then decrease, and reached the maximum value of 35% when the aging temperature was 620 °C [13]. The law and mechanism of austenite reverse phase transformation of 0Cr13Ni4Mo maraging stainless steel during different tempering processes were studied by Li D.Z. et al., revealing that Ni and Mn elements can be enriched through uphill diffusion, promoting the occurrence of austenite reverse phase transformation. Moreover, when the alloy was tempered at a tempering temperature above 600 °C, the content of reversed austenite at room temperature reached up to 22.21% [14]. From the above-reported results, it can be seen that for 04Cr13Ni5Mo maraging stainless steel, slight changes in heat treatment process parameters would affect the content of reversed austenite in the structure, indicating that the evolution law as well as the final content of reversed austenite in the microstructure of the alloy have a high sensitivity to the heat treatment process. How to obtain the required mechanical properties through heat treatment and ensure the uniformity of the properties of large forgings has become the focus of research and an urgent problem to be solved [15]. In order to master the key microstructure control law of the heat treatment process of 04Cr13Ni5Mo maraging stainless steel, it is necessary to systematically clarify the evolution law of the microstructure and mechanical properties of the material under different heat treatment processes.

Based on the above background, taking 04Cr13Ni5Mo maraging stainless steel as the research object, this study systematically studied the evolution law of the microstructure and mechanical properties under different tempering temperatures. Furthermore, characterization techniques such as electron backscatter diffraction (EBSD) and electron probe microanalysis (EPMA) were adopted to explore the change law of reversed austenite. While enriching and improving the microstructure regulation theory, this study can also lay an experimental and theoretical foundation for the in-depth exploration of the mechanical property potential of 04Cr13Ni5Mo maraging stainless steel.

## 2. Experimental Conditions and Methods

### 2.1. Materials Composition

The experimental material selected in this study was the typical 04Cr13Ni5Mo maraging stainless steel, the alloy composition of which was designed and smelted with reference to the standard requirements of the composition of large impact turbine runner forgings reported in the literature. The specific alloy composition is shown in Table 1 [8]. In the process of composition design, the equivalent ratio of Ni element to Cr element higher than 0.42 is a sufficient condition to ensure that the volume fraction of high-temperature δ ferrite in this type of alloy steel is as low as possible [1,16]. After calculation, the Ni/Cr equivalent ratio of the 04Cr13Ni5Mo maraging stainless steel used in this study was 0.51. The tested steel was smelted in a 150 kg vacuum induction furnace, then forged into a forged billet with a cross-sectional size of 120 × 120 mm, and after machining, cut into square billets with a size of 60 × 60 × 60 mm for the subsequent heat treatment experiments.

### 2.2. Equilibrium Transformation Law of the Tested Steel

**Figure 1:**
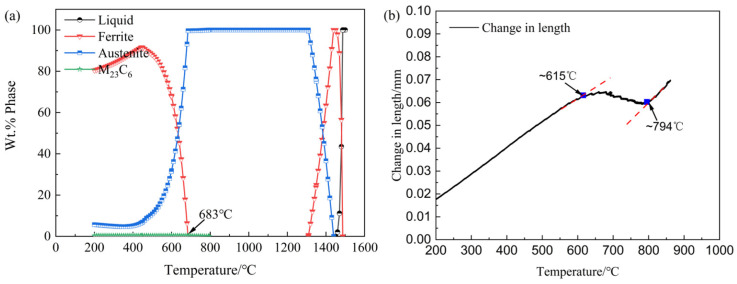
(**a**) Equilibrium transition phase diagram of the studied steel and (**b**) thermal expansion curve of the studied alloy obtained through thermal dilatometer.

In order to master the phase equilibrium transformation law of 04Cr13Ni5Mo maraging stainless steel and evaluate its hardenability, so as to provide a theoretical reference for the subsequent formulation of the heat treatment process, JMatPro 7.0 thermodynamic calculation software was used to calculate the equilibrium transformation phase diagram of the tested steel, and the calculation results are shown in Figure 1a. According to the calculation results, among the critical phase transformation temperatures Ac1 and Ac3 of 04Cr13Ni5Mo, Ac1 was relatively difficult to determine, while Ac3 was about 683 °C. In order to further determine the critical phase transformation temperature of the test steel, a critical temperature test was conducted on the L78 RITA fully automatic phase transformation instrument (Linseis Messgeraete GmbH, Selb, Germany). The sample with the size of φ3 × 10 mm was heated from room temperature to 870 °C at a heating rate of 0.01 °C/s, and the relationship curve between the dimensional change in the tested steel, the test temperature, and time during the heating process was obtained. Figure 1b shows the thermal expansion curve of the tested 04Cr13Ni5Mo maraging stainless steel. It can be seen from the test results that the Ac1 and Ac3 temperatures of the tested steel were about 615 °C and 794 °C, respectively. The difference between the measured Ac3 and the JMatPro-calculated Ac3 likely arose from kinetic effects during continuous heating and the limitations of the thermodynamic database for this alloy system. In addition, according to the phase equilibrium transformation diagram, the formation law of M23C6 intermetallic compounds, which are closely related to the mechanical properties of the material, can be observed. The ideal formation temperature of this intermetallic compound during the cooling process was about 793 °C.

### 2.3. Experimental Method

#### 2.3.1. Heat Treatment Experiment Process

**Figure 2:**
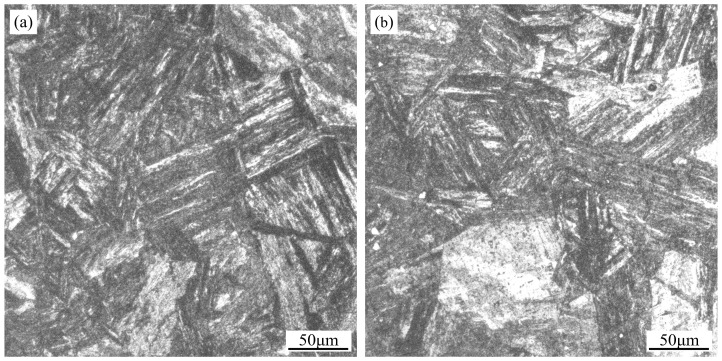
Optical micrograph of the studied steel processed by air cooling. (**a**) selected area 1; (**b**) selected area 2.

Based on the equilibrium transformation phase diagram and the thermal expansion curve of the tested steel, it was determined that austenitization at 1000 °C could guarantee that the primary intermetallic compounds in the alloy were fully dissolved in the solid solution. Accordingly, 1000 °C was selected as the austenitization temperature for this study. To evaluate the hardenability of the tested steel, the cubic specimens with dimensions of 60 × 60 × 60 mm were first heated to 1000 °C at a rate of 10 °C/min, held at this temperature for 1 h, and then air-cooled. The microstructure of the heat-treated specimen was characterized via optical microscopy. Figure 2 presents the optical micrograph of the tested 04Cr13Ni5Mo maraging stainless steel after air cooling. From the metallographic structure, it was observed that under this condition, the microstructure contained regions composed of a series of parallel block-like structures. Combined with the alloy composition of 04Cr13Ni5Mo, it was confirmed that the microstructure was dominated by lath martensite and block martensite. In addition, according to the existing literature, the δ ferrite structure, which is distributed in strips along the austenite grain boundaries, is readily observable in the microstructure of this type of alloy after heat treatment [1]. Combined with the observations in Figure 2, no obvious δ ferrite structure was detected in the microstructure of the smelted 04Cr13Ni5Mo maraging stainless steel after austenitization and air-cooling treatment, indicating that the δ ferrite structure was effectively suppressed. Combined with the above results and the conclusions reported in the literature [16], it was demonstrated that the tested steel exhibited excellent hardenability. Specifically, a fully martensitic microstructure could be obtained under air-cooling conditions, which achieved the desired quenching effect.

**Figure 3:**
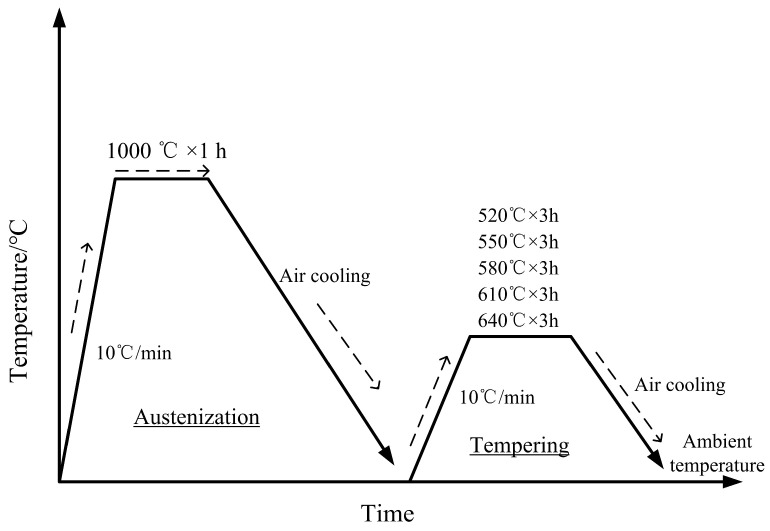
Scheme of heat-treatment experiment.

A quenching and tempering process was adopted for the heat treatment experiments of the tested steel. During the quenching process, the tested steel was heated to 1000 °C at a rate of 10 °C/min, held for 1 h for austenitization, and then air-cooled to room temperature. Subsequently, the quenched specimens were reheated to the temperature range of 520–640 °C (with an interval of 30 °C) at a heating rate of 10 °C/min, tempered at the target temperature for 3 h, and then air-cooled. The schematic of the specific heat treatment process is shown in Figure 3, and the heat treatment experiments were conducted on a KBF-1200C-L muffle furnace.

#### 2.3.2. Microstructure Characterization

After the completion of the heat treatment experiments, the microstructure of the 04Cr13Ni5Mo maraging stainless steel obtained at different tempering temperatures was characterized. The square specimens were cut along the longitudinal section of the cross-section for sample preparation. After mechanical grinding and polishing, the specimens were etched with 4% nitric acid alcohol solution (etching time: 30 s) for surface morphology observation. Before EBSD and XRD tests, the surface stress layer of the specimens was removed by electrolytic polishing with 13% perchloric acid alcohol solution to avoid the reverse austenite transformation caused by strain (voltage 20 V, time 20 s). The surface morphology observation and EBSD testing were performed on a field emission scanning electron microscope (Model: FEI Quanta 600, FEI, Hillsboro, OR, USA). The EBSD test was conducted at an accelerating voltage of 20 kV, with a working distance of 15 mm and a sample tilt angle of 70°. The test step size was 0.05 μm. The indexing success rates were all higher than 95%. And the post-processing of the EBSD test results was carried out using Channel 5 software. It should be noted that while EBSD provides valuable information on phase distribution and crystallographic orientation, the quantification of phase fractions by EBSD is sensitive to surface preparation and local microstructure variations. Therefore, XRD analysis was employed for bulk phase quantification using a D8 ADVANCE diffractometer (Bruker, Billerica, MA, USA) with a Cu target. (see Section 3.2). The parameters were as follows: scan range 40.0–96°, step size 0.04°, time per step 1 s, accelerating voltage 45 kV, tube current 200 mA. For EPMA analysis, the mechanically ground and polished specimens were etched with 4% nitric acid alcohol solution for 20 s.

#### 2.3.3. Mechanical Property Testing

After the completion of the heat treatment experiments, the tensile properties and room-temperature impact toughness properties of the 04Cr13Ni5M obtained at different tempering temperatures were tested. All test specimens were extracted from the central region of square samples with dimensions of 60 × 60 × 60 mm, with their longitudinal axes oriented parallel to the principal forging direction. The preparation of tensile specimens and the corresponding property tests were conducted in accordance with the standard Metallic materials—Tensile testing—Part 1: Method of test at room temperature (GB/T 228.1-2021 [17]). Round bar specimens were adopted for the tests, with the diameter of the parallel section and the gauge length selected as 5 mm and 25 mm, respectively. According to GB/T 229-2020 [18], the impact toughness test was conducted using Charpy U-notch specimens with a size of 10 × 10 × 55 mm. The tensile test and impact property test were performed on a 100 kN electronic universal material testing machine (Model: BOYI 2025-010) and a pendulum impact testing machine, respectively. Under each heat treatment condition, three tensile tests and three impact tests were conducted. The mechanical property results were presented in the form of a mean value, and the error line represents the standard deviation.

## 3. Results and Discussion

### 3.1. Microstructure Characteristics at Different Tempering Temperatures

**Figure 4:**
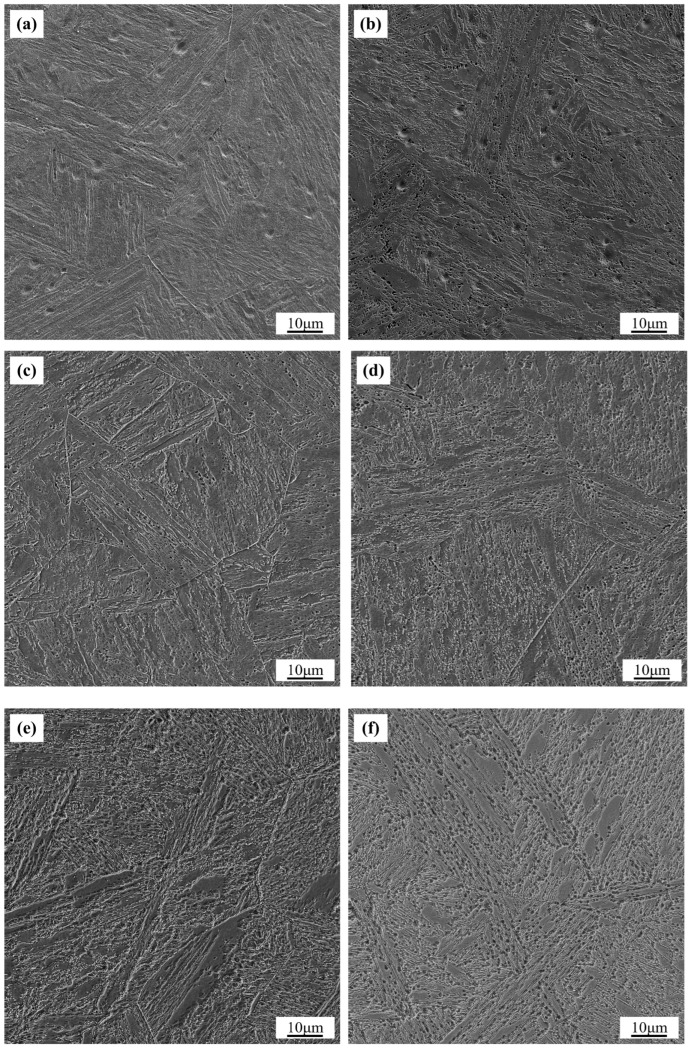
Morphology of studied steel processed by different tempering temperatures: (**a**) quenching, (**b**) 520 °C, (**c**) 550 °C, (**d**) 580 °C, (**e**) 610 °C, (**f**) 640 °C.

Figure 4 presents the surface morphology photographs of the tested steel after different quenching and tempering processes. As can be seen from Figure 4a, under air-cooled conditions after quenching, the surface protrusions of the slat structure and the blocky protrusion structure can be observed. Combined with Figure 2, it was confirmed that these structures were lath martensite and block martensite. After tempering at 520 °C, the lath relief phenomenon on the surface of the tested steel was obvious, and the long strip and block ferrite matrix structure could be observed in the surface morphology. The overall microstructure of the tested steel presented the typical characteristics of tempered sorbite. As the tempering temperature gradually increased from 520 °C to 640 °C, the morphology of the tested steel also presented the typical characteristics of tempered sorbite, while the lath morphology was weakened, and the block and lath ferrite matrix could be observed on the surface of the tested steel, as shown in Figure 4b–f.

### 3.2. Crystallographic Orientation Characteristics at Different Tempering Temperatures

**Figure 5:**
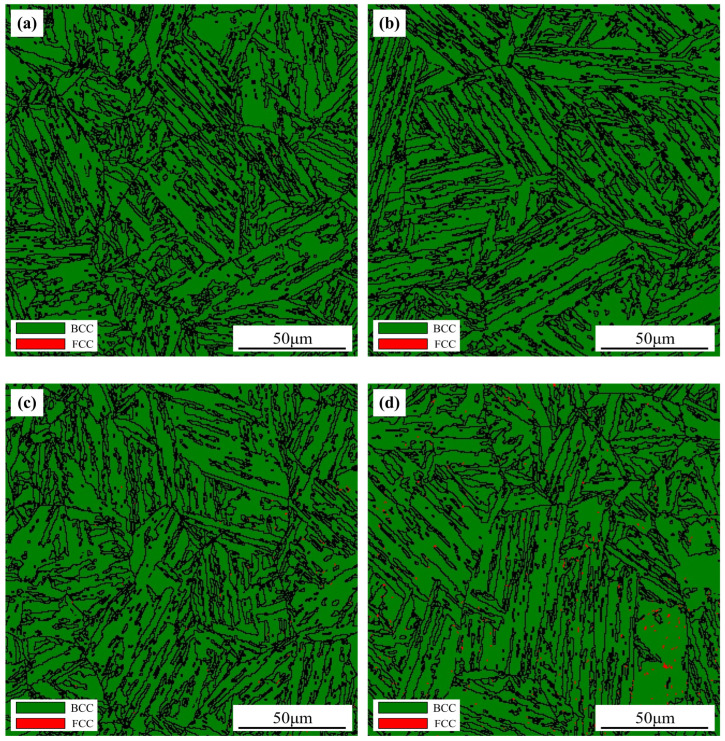

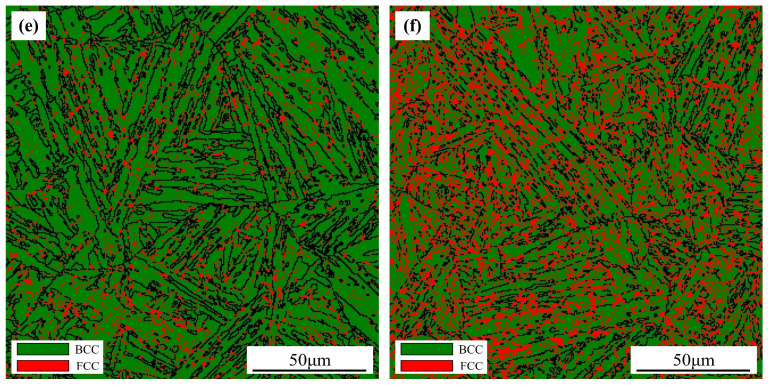
Phase diagram of studied steel: (**a**) quenching, (**b**) 520 °C, (**c**) 550 °C, (**d**) 580 °C, (**e**) 610 °C, (**f**) 640 °C.

**Figure 6:**
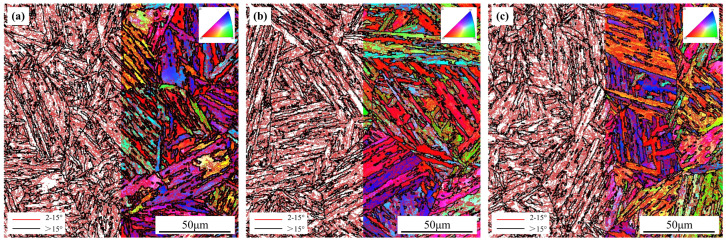

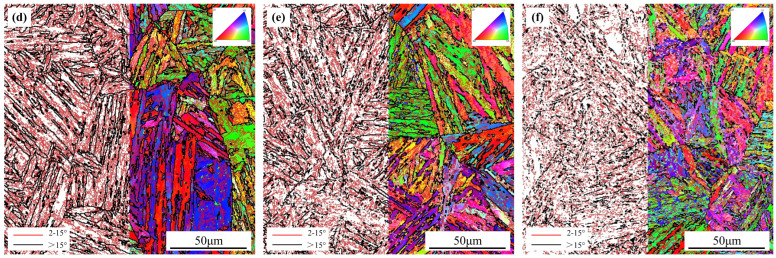
The grain boundary map and IPF figure of the studied steel processed at different tempering temperatures: (**a**) quenching, (**b**) 520 °C, (**c**) 550 °C, (**d**) 580 °C, (**e**) 610 °C, (**f**) 640 °C.

**Figure 7:**
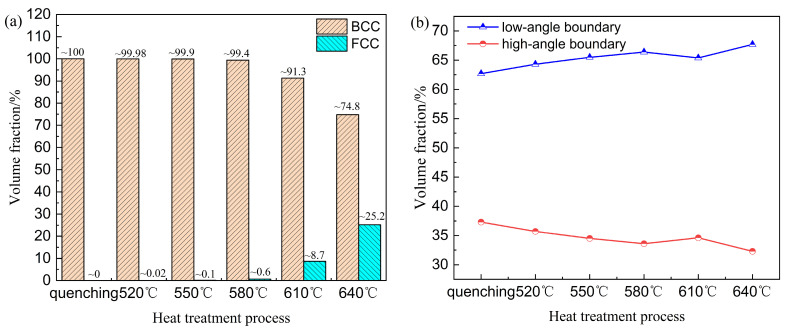
Statistics of crystallographic orientation of studied alloy processed at different tempering temperatures: (**a**) proportional information of phase obtained from EBSD analysis, (**b**) distribution feature of grain boundary.

In order to explore the crystallographic orientation information of test steels at different tempering temperatures, a systematic study was conducted on the EBSD test results of test steels, and the results are shown in Figure 5, Figure 6 and Figure 7.

From the analysis results of the phase diagram in Figure 5, it was observed that in the quenched state, the phases in the microstructure were mainly of BCC crystal structure, as shown in Figure 5a. After quenching and tempering treatment, when the tempering temperature was 520 °C and 550 °C, the phase with FCC crystal structure, which was distributed in fine dots at some grain boundaries in the microstructure, could be detected, as shown in Figure 5b,c. When the tempering temperature increased to 580 °C, the volume fraction of the phase with FCC crystal structure distributed in dots in the structure of the tested steel increased slightly, as shown in Figure 5d. When the tempering temperature further increased to 610 °C, the proportion of the phase with the FCC crystal structure in the tested steel increased significantly. In addition to the dot-like FCC phase, the long strip and spindle-shaped FCC phase could be observed in the structure of the tested steel, as shown in Figure 5e. When the tempering temperature further increased to 640 °C, the proportion of the phase with the FCC crystal structure in the tested steel increased significantly. Most of the FCC phases were distributed along the grain boundaries or subgrain boundaries, as shown in the red-marked area in Figure 5f.

To clarify the change law of the proportion of high-angle grain boundaries (misorientation ≥ 15°) and low-angle grain boundaries (misorientation < 15°) in the tested steel with the increase in tempering temperature, the grain boundary map and IPF figure of the specimens in the quenched state and different tempered states were systematically characterized, and the results are shown in Figure 6. From the grain boundary distribution map, it was observed that in the tested steel in the quenched state and the tempered states at 520 °C, 550 °C, and 580 °C, the grain boundaries of each martensite region outlined by high-angle grain boundaries were clearly visible, and the high-angle grain boundaries were distributed in the form of continuous grain boundaries, as shown in Figure 6a–d. In contrast, in the tested steel tempered at 610 °C and 640 °C, the continuity of the grain boundaries of each martensite region outlined by high-angle grain boundaries decreased. The high-angle grain boundaries were distributed in a discontinuous manner under the original distribution trend of continuous grain boundaries, as shown in Figure 6e,f. Combined with the aforementioned phase ratio distribution map, it was found that a certain amount of FCC phase appeared at the grain boundaries of the tested steel tempered at 610 °C and 640 °C, indicating that the formation of FCC phase led to the discontinuity of high-angle grain boundaries.

To quantitatively describe the change trend of different phase ratios and the ratio of high-angle and low-angle grain boundaries in the tested steel with tempering temperature, the phase ratio information and the ratio information of high-angle and low-angle grain boundaries were statistically analyzed, and the results are shown in Figure 7. From the statistical results, it was found that the proportion of FCC phase in the tested steel under the quenched state and the tempered states at 520 °C, 550 °C, and 580 °C was almost negligible, indicating that under such heat treatment conditions, the contents of retained austenite or reversed austenite in the tested steel were almost 0. However, when the tempering temperature increased to above 610 °C, the percentage content of reversed austenite in the tested steel increased significantly.

In the tempered state at 610 °C, the content of reversed austenite in the tested steel increased to 8.7%, while in the tempered state at 640 °C, the content of reversed austenite reached the maximum value of 25.2% (as measured by EBSD), indicating that the content of reversed austenite with FCC crystal structure changed significantly after the tempering temperature increased to a certain extent. In comparison, the change in the proportions of high-angle and low-angle grain boundaries presented an almost linear trend with with increasing tempering temperature. In the quenched state, the proportion of low-angle grain boundaries was about 62.7%. As the tested steel transformed from the quenched state to the tempered state, the proportion of low-angle grain boundaries increased. After tempering at 520 °C, the proportion of low-angle grain boundaries and high-angle grain boundaries in the tested steel were 64.3% and 35.7%, respectively. As the tempering temperature increased from 520 °C to 640 °C, the proportion of low-angle grain boundaries increased from 64.3% to 67.7%. It is worth noting that normally, the proportion of low-angle grain boundaries in the microstructure of alloy steel will decrease with the reduction in dislocation density during the tempering process. However, for the 04Cr13Ni5Mo, with the increase in tempering temperature, the proportion of low-angle grain boundaries presented an increasing trend, which was mainly related to the formation of reversed austenite during the tempering process. With the increase in tempering temperature, the reversed austenite formed attached to the original high-angle grain boundaries would reorganize the high-angle grain boundaries in martensite into the low-angle grain boundaries of reversed austenite, leading to the decrease in the proportion of high-angle grain boundaries. Moreover, this reduction was greater than the reduction in small-angle grain boundaries affected by tempering, ultimately resulting in the phenomenon that the proportion of small-angle grain boundaries increases with the rise in tempering temperature.

**Figure 8:**
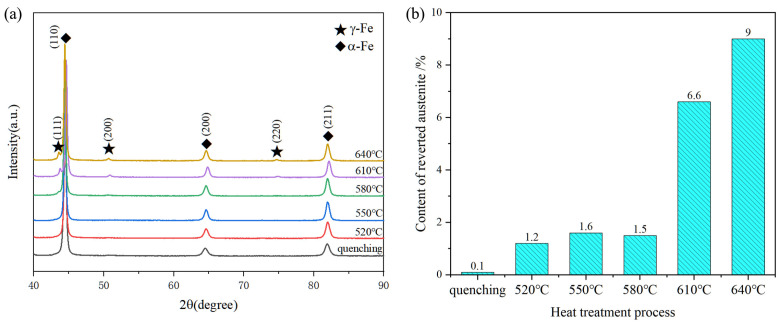
XRD results of the test steel at different tempering temperatures: (**a**) diffraction patterns, (**b**) fraction of reverse austenite.

Considering the limited measurement area of EBSD and its high sensitivity to testing parameters and local microstructure variations, a more accurate quantification of reverted austenite content was achieved by applying XRD combined with Rietveld full-pattern refinement for phase analysis over a macroscopic region. The results are presented in Figure 8. As XRD analysis (Figure 8) provides a more reliable quantitative assessment, the EBSD results are primarily used here to illustrate the spatial distribution and morphological evolution of reversed austenite, rather than for precise quantification. A comparison between the XRD and EBSD results revealed a consistent increasing trend in reverted austenite content with rising tempering temperature. At a tempering temperature of 610 °C, the reverted austenite content exhibits a sharp increase to 6.6%. Notably, for the sample tempered at 640 °C, the EBSD measurement (25.2%) was considerably higher than the XRD result (9%). According to Ref. [19], EBSD is highly sensitive to surface preparation and the specific region selected for analysis. In contrast, for FCC phases precipitated along grain or subgrain boundaries, XRD measurements can circumvent the randomness associated with local area selection and thus more reliably reflect the actual phase composition within the bulk volume of the material.

### 3.3. Mechanical Properties at Different Tempering Temperatures

**Figure 9:**
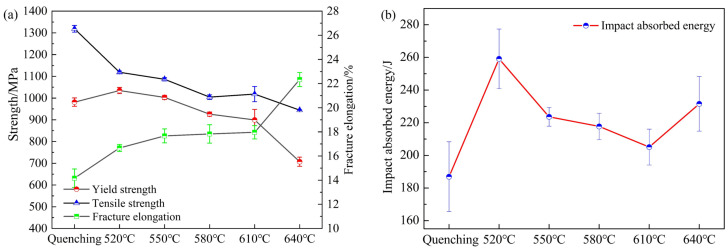
Distribution of mechanical properties processed by different tempering treatments: (**a**) tensile properties, and (**b**) impact properties at room temperature.

In order to explore the variation law of the strengthening and toughening properties under different tempering temperatures, the mechanical properties of the tested steel in different states were analyzed, and the results are shown in Figure 9. It can be known from the distribution law of mechanical properties that in the quenched state, the average tensile strength of the tested steel reached the maximum value of 1318.7 MPa, and the average yield strength was at a medium level of 981.3 MPa. The plasticity and toughness were relatively low under this condition, with the average elongation after fracture of 14.17%, and the room-temperature impact absorption energy of 186.9 J.

Compared with the quenched state, the overall performance became superior after the application of the tempering process. Specifically, the tensile strength of the tested steel decreased, the yield strength increased, and the plasticity and toughness were significantly improved. As the tempering temperature increased from 520 °C to 580 °C, the tensile strength and yield strength of the tested steel both presented a decreasing trend, and the elongation after fracture increased, while the impact absorption energy presented a decreasing trend, as shown in Figure 9.

As the tempering temperature increased from 580 °C to 640 °C, the tensile strength and yield strength of the tested steel first remained stable and then decreased, while the elongation after fracture and impact absorption energy first remained stable and then increased significantly. Based on the above performance variation patterns, it can be known that under the tempering condition of 610 °C, the strength, plasticity, and toughness of the tested steel achieved the optimal matching. Tempering at this temperature, the average yield strength and average tensile strength of the tested steel were 899.3 MPa and 1018.3 MPa, respectively, and the average elongation after fracture and average room-temperature impact absorption energy were 18.0% and 205.1 J, respectively. Under the tempering condition of 640 °C, the tested steel presented the characteristics of relatively low strength, good plasticity, and toughness. The average yield strength and average tensile strength of the tested steel were 707.3 MPa and 944.3 MPa, respectively, and the average elongation after fracture and average room-temperature impact absorption energy were ~22.2% and ~231.6 J, respectively.

### 3.4. Evolution Law of Reversed Austenite and Strengthening and Toughening Mechanism at Different Tempering Temperatures

Generally, for maraging hardened stainless steel, the content of reversed austenite in the microstructure is crucial to its mechanical properties. On the one hand, with martensite characterized by high-density dislocations as the matrix structure, combined with the precipitation of various intermetallic compounds during the tempering process, the high strength of the alloy can be guaranteed through the coupling of different strengthening mechanisms, such as dislocation strengthening, grain refinement strengthening, and second-phase precipitation strengthening. On the other hand, by adopting different heat treatment processes, a certain amount of metastable reversed austenite structure can be introduced into the martensite matrix structure, and the strength and plasticity of the alloy steel can be improved through the TRIP effect mechanism [19]. As a typical grade of maraging precipitation hardening stainless steel, 04Cr13Ni5Mo also follows the above strengthening and toughening theory.

Combined with the above microstructure evolution law and the mechanical property distribution characteristics, it can be found that in the quenched state, the content of retained austenite in the structure of the tested steel was almost negligible. In this condition, the high-density dislocations on the martensite lath substructure in the tested steel were the key to ensuring the strength of the alloy, and the average tensile strength of the alloy reached 1318.7 MPa. However, the presence of a higher dislocation density can cause the easy entanglement of dislocations during plastic deformation, and the mutually entangled dislocations tend to interweave with each other, causing local stress concentration, resulting in non-uniform plastic deformation of the alloy and deterioration of the material’s plasticity. In this state, the average elongation after fracture of the alloy was approximately 14.17%. After different temperature tempering treatments performed on the quenched specimens, the alloy matrix structure transformed into a tempered sorbite structure, and the dislocation density decreased. The formation of a sufficient amount of reversed austenite in the structure primarily depends on whether the tempering temperature falls within the two-phase region (ferrite + austenite) of the tested steel. From the critical phase transformation temperature of the tested steel measured in Section 2.2, it could be found that in the range of 520 °C to 580 °C, the tested steel had not entered the two-phase region of ferrite and austenite. Within this tempering temperature range, some residual austenite had improved stability due to the diffusion of elements such as C and existed in a point-like form. When the tempering temperature entered the two-phase region, the reversed austenite formed on the ferrite matrix would stabilize due to the enrichment of elements such as Ni and Mn, resulting in the formation of a large amount of reversed austenite in the final room-temperature structure.

**Figure 10:**
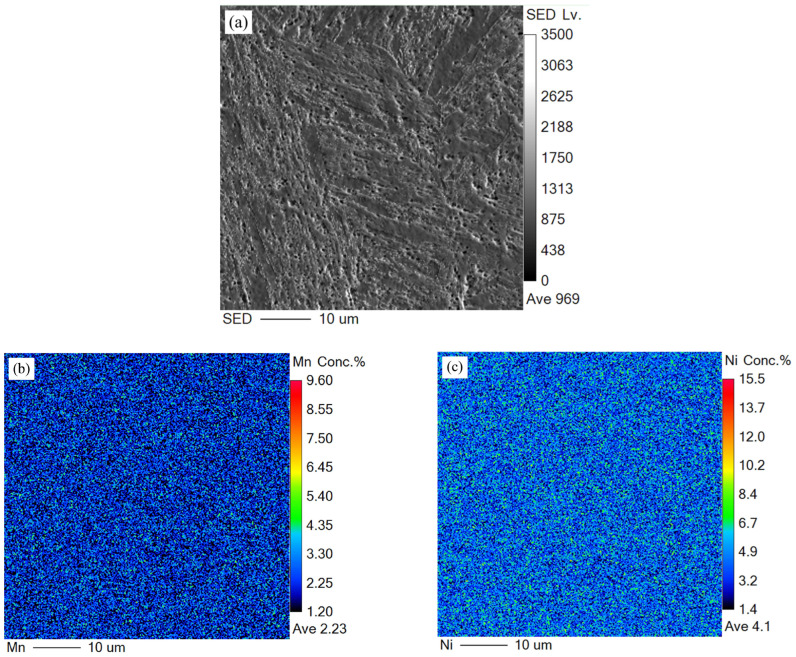
Surface scanning map of the studied steel processed by 580 °C tempering obtained through EPMA detection. (**a**) SED map, (**b**) Mn element, (**c**) Ni element.

**Figure 11:**
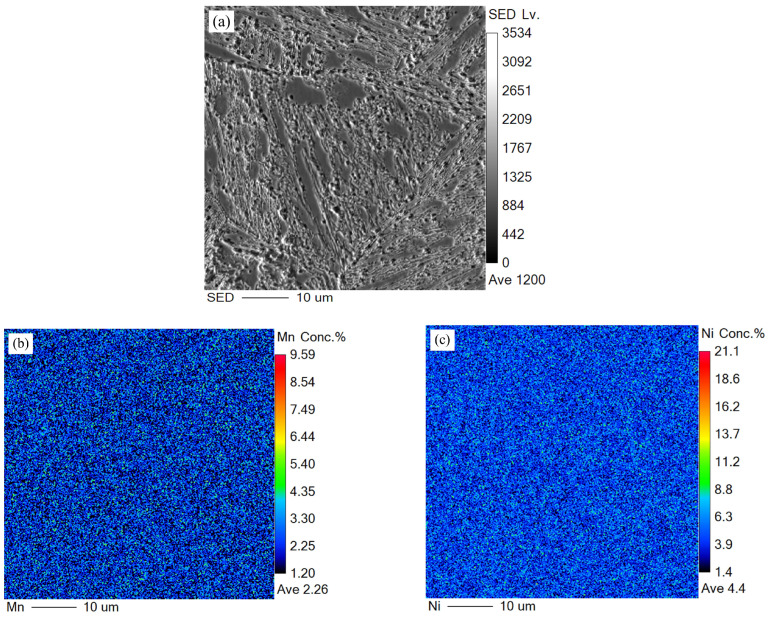
Surface scanning map of the studied steel processed by 640 °C tempering obtained through EPMA detection. (**a**) SED map, (**b**) Mn element, (**c**) Ni element.

For the 04Cr13Ni5Mo maraging stainless steel, some reported research results show that the enriched Mn and Ni elements are the main elements that stabilize austenite [14,20,21]. To verify the types of stabilizing elements of reversed austenite in this study, the surface element distribution of the tested steel at two typical tempering temperatures of 580 °C and 640 °C was comparatively analyzed. The analysis results of the surface element distribution of the tested steel obtained by EPMA detection are shown in Figure 10 and Figure 11. During the test process, the distributions of Mn and Ni were mainly analyzed. As can be seen from Figure 10, in the tempered state of 580 °C, the distributions of the Mn element and the Ni element were relatively uniform. The average strengths of Mn and Ni elements were 2.23 and 4.1, respectively, and the maximum local strengths of the three elements reached 9.6 and 15.5. Similarly, the distribution of Mn and Ni elements on the surface of the tested steel in the tempered state at 640 °C was analyzed, and the results are shown in Figure 11. It could be found that the average intensity of Mn and Ni were 2.26 and 4.4, respectively, which were basically consistent with the selected area of the 580 °C tempered sample, as there was no obvious segregation observed in this area. However, in this area, the maximum local intensities of these two elements reached 9.59 and 21.1, respectively. By comparing the surface element distribution law of the tested steel in the tempered state at 580 °C and 640 °C, it was revealed that the distribution law of Mn on the surface of the tested steel in the tempered state at 640 °C was similar. However, the average value of Ni element distribution and the local peak value of Ni element were both higher than those in the tempered state at 580 °C, indicating that the enrichment of Ni element has a high correlation with the precipitation and stability of reverse austenite, which was consistent with the results reported in the aforementioned literature [22,23]. For the tested 04Cr13Ni5Mo maraging stainless steel, when the tempering temperature is higher than 610 °C, a certain amount of reversed austenite will be formed at the grain boundary positions of the ferrite matrix in the form of nucleation and growth. During the holding process, the Ni element will be enriched into the reversed austenite in the form of uphill diffusion, reducing the martensite start temperature and improving the stability of reversed austenite. After the tempering and holding process is completed, the reversed austenite, due to its enhanced stability, can be retained when cooled to room temperature, improving the strength and toughness of the alloy.

For the 04Cr13Ni5Mo maraging stainless steel used in this experiment, when the tempering temperature was 610 °C, the microstructure was composed of tempered sorbite and a certain amount of reversed austenite (about 6.6%). Under the coupling effect of grain refinement strengthening, dislocation strengthening, second phase strengthening, and the TRIP effect of reversed austenite, the strength, plasticity, and toughness of the tested steel achieved the optimal matching. The average yield strength was 899.3 MPa, the average tensile strength was 1018.3 MPa, and the average elongation after fracture and average room-temperature impact absorption energy were respectively 18.0% and 205.1 J. As the tempering temperature increased to 640 °C, the effect of dislocation strengthening in the strengthening mechanism of the tested steel weakened, while the TRIP effect caused by 9.0% reversed austenite was significantly enhanced. Consequently, the tested steel presented the characteristics of low strength, high plasticity, and toughness. The average elongation after fracture of the tested steel reached 22.2%, and the average room-temperature impact absorption energy reached 231.6 J.

## 4. Conclusions

With the evolution law of microstructure and mechanical properties of the 04Cr13Ni5Mo maraging stainless steel at different tempering temperatures systematically investigated in this study, the main conclusions can be summarized as follows:

(a) The microstructure of the tested steel was dominated by lath martensite and block martensite in the quenched state. With the increase in tempering temperature, the microstructure gradually transformed into tempered sorbite. When the tempering temperature increased to above 610 °C, the content of reversed austenite increased significantly, reaching 6.6% at 610 °C and the maximum value of 9.0% at 640 °C as determined by XRD analysis.
(b) Under the treatment process of austenitization at 1000 °C for 1 h and tempering at 610 °C for 3 h, the strength, plasticity, and toughness of the tested 04Cr13Ni5Mo achieved the optimal matching. The average yield strength was 899.3 MPa, the average tensile strength was 1018.3 MPa, and the average elongation after fracture and average room-temperature impact absorption energy were 18.0% and 205.1 J, respectively.
(c) The enrichment of the Ni element plays a significant role in the formation and stability of the reverted austenite in 04Cr13Ni5Mo stainless steel. As the tempering temperature increased from 580 °C to 640 °C, the Ni element was enriched in the reversed austenite through uphill diffusion, and the local maximum value of Ni on the surface of the test steel increased from 15.5 to 21.1.

## Figures and Tables

**Table 1:** Chemical composition of the studied 04Cr13Ni5Mo stainless steel (mass fraction, %).

Element	C	Si	Mn	Cr	Ni	Mo	V	P	S
Content (wt.%)	0.04	0.37	0.69	12.7	4.51	0.62	0.05	0.012	0.37

Nieq = ω(Ni) + 30ω(C + N) + 0.5ω(Mn); Creq = ω(Cr + Mo) + 1.5ω(Si).

## Data Availability

The original contributions presented in this study are included in the article. Further inquiries can be directed to the corresponding authors.
